# Oncoprotein metastasis and its suppression revisited

**DOI:** 10.1186/1756-9966-29-30

**Published:** 2010-04-09

**Authors:** Razvan T Radulescu

**Affiliations:** 1 Molecular Concepts Research (MCR), Muenster, Germany

## Abstract

The past two decades have witnessed an increasing appreciation of the role of the tumor microenvironment, of genetic and epigenetic alterations in normal cells adjacent to tumors and of the migration of normal cells with aberrant intrinsic properties in cancer pathophysiology. Aside from these insights, a novel concept termed "oncoprotein metastasis" (OPM) has recently been advanced and proposed to reflect protein-based neoplastic phenomena that might occur even before any modifications relating to the morphology, location or (epi)genetic outfit of cells during the malignant process. Here, evidence is presented that supports the OPM perception and thus should contribute not only to further rethink the definition of a normal cell, but also the treatment of cancer disease in the years to come.

## Background

Over the past decades of molecular cancer research, many investigators have strived to understand the single subcellular alterations that make a normal cell switch to become a cancer cell. One of the first key advances along these lines was the detection of a minute chromosome in chronic myelogenous leukemia cells [1]. Subsequently, many more aberrant chromosomes resulting from chromosomal alterations such as translocations and deletions were identified in various malignant diseases, mainly affecting the hematological lineage. A corollary of this view on a chromosomal origin of neoplasias was the postulate according to which cancer arises from chromosomal aberrations occurring in single cells that, due to these pathological subcellular changes, start proliferating in a clonal fashion giving rise to macroscopic tumors [2]. Historically intersecting with this perception was the uncovering in normal DNA of cellular oncogenes resembling their viral counterparts [3] which marked the beginning of the (proto)oncogene paradigm in cancer research according to which (amplified) oncogenes drive cancer cell proliferation. On the other hand, alterations in a second class of genes, more specifically partial or complete losses of tumor suppressor genes in tumor cells [4] and, as was found a number of years later, also in (morphologically) normal cells adjacent to primary tumors [5] were equally recognized as paramount in the pathogenesis of neoplasias.

These chromosomal and genetic alterations as well as aneuploidic sets of chromosomes are widely believed until nowadays to underlie the neoplastic transformation of normal cells into morphologically overt cancer cells although a recent re-evaluation of this aspect has revealed that aneuploidy can under certain conditions have also the opposite effect of tumor suppression [6]. Notwithstanding these significant leaps in our knowledge on cancer, this disease remained largely undefeated by the end of the 1990s [7] and has stayed so in its metastatic form until even today, despite recent drug achievements such as herceptin and imatinib that each target the product of an altered oncogene. The reason why a genuine therapeutic breakthrough remains as yet unachieved [8] could likely be that our strategies to tackle cancer are still incompletely integrating the many pieces of the puzzle that we have already accumulated and the various concepts already advanced on the basis of this knowledge.

In accordance with this interpretation, Richmond Prehn asked already in 1994 [9] the crucial hen-and-egg question on cancer pathogenesis as to what comes first: the cancer process per se or the mutations in genes pertaining to morphologically overt cancer cells? This call for a possible paradigm shift remains a challenge until today, yet some key elements of such malignant process can be already found in the literature of the past two decades. Accordingly, it has been observed that the cancer process may begin very early, specifically at the level of the DNA structure in (morphologically) normal cells adjacent to primary tumors [10]. Furthermore, it was concluded that certain post-translational events that inactivate a given tumor suppressor protein could be regarded as functionally equivalent to an inactivating mutation of its gene, for instance retinoblastoma protein (RB)'s physical interaction with a viral oncoprotein or the former's hyperphosphorylation [11]. Post-translational events such as the increase in the stability of an oncoprotein were equally recognized as crucial for a pathologically accelerated cell cycle progression [12]. Moreover, it was found that hypermethylations in the promoters of genes encoding growth-suppressive proteins often mimic the patterns for mutations in the respective genes [13]. Also, the phenomenon of nuclear exclusion of tumor suppressors through their cytoplasmic sequestration by distinct proteins has been recognized as another mechanism corresponding to an inactivating mutation of the respective tumor suppressor gene [14,15]. In addition, protein-based inflammatory processes in the tumor microenvironment are likely to influence the tumor cells embedded in that specific area [16]. The key twist common to these molecular insights is that the post-translational/epigenetic events they refer to may conceivably occur in morphologically normal cells that, moreover, have not yet acquired modifications in their growth-regulatory genes, yet these events might already constitute a (pre)malignant process that is ongoing in these seemingly normal cells.

## Oncoprotein metastasis disjoined: a reappraisal

**Figure 1:**
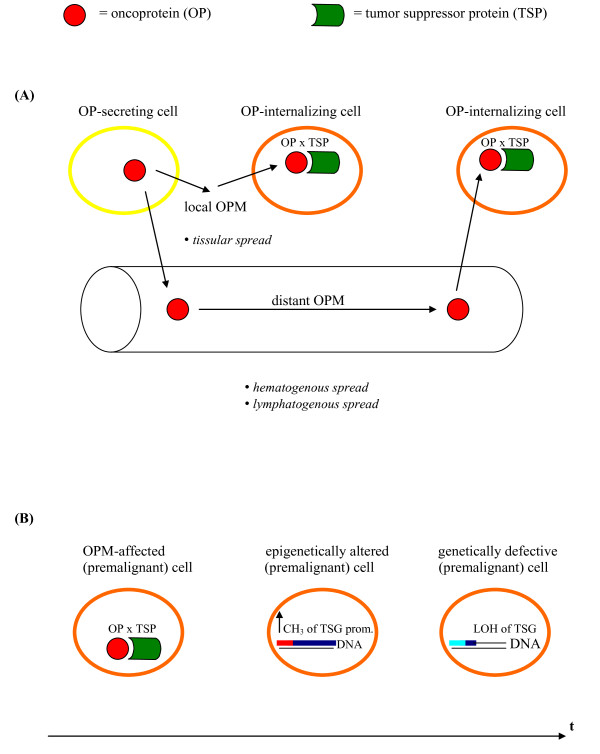
**Schematic definition of the process of oncoprotein metastasis (OPM) accompanied by physical interactions between oncoproteins (OPs) and tumor suppressor proteins (TSPs)**: a) spatially, consisting in the *local, tissular penetration *of OPs into cells adjacent to the cells from which the OPs originate (thereby extending the paracrine principle) and/or their *systemic spread *via blood and lymphatic vessels to distant tissues/organs (thereby extending the endocrine principle), each of which would be ensued by (e.g. nucleocrine [28,31]) OP-TSP complex formations (OP × TSP); it should be also stated here that the OP-secreting cells are not necessarily tumor cells, but could be normal cells, e.g. pancreatic β-cells that secrete (excessive amounts of) insulin in response to (blood-borne) tumoral stimuli and thus cause a well-known (cancer-associated) state of hyperinsulinemia; b) temporally, consisting in the OPM-associated and carcinogenesis-initiating event of OP-TSP complex formations (OP × TSP) that *precede *the epigenetic silencing of the corresponding tumor suppressor gene-caused by the hypermethylation of its promoter-which in turn is *subsequently functionally mimicked *by a loss of heterozygosity (LOH) of the same gene, all of which changes would occur in (morphologically) normal, yet likely premalignant cells.

Several years ago, I have expanded this view by my concept on an oncoprotein metastasis (OPM) and its possible therapeutic reversal [17,18]. In analogy to the possibilities of a transfer of disease from one organ site to another, i.e. of metastasis by means of a) *microorganisms*, e.g. bacteria [19], or b) *cells*, e.g. (morphologically) malignant cells [20] or, as shown after the above-mentioned oncoprotein metastasis concept had been advanced, even (morphologically) normal/untransformed cells [21], I have proposed a third mechanism (Fig. 1a) according to which growth-promoting *proteins *such as insulin that are known to be capable of translocating across cellular membranes may equally convey, if present in abnormal tissue concentrations, initial pathologic signals to proximal and distant tissues and thus contribute to their malignant transformation prior to the occurrence of any (epi)genetic and/or chromosomal alterations [17,18]. Thereby, I had also surmised that defective tumor-suppressive mechanisms in such OPM-affected tissues would partly account for the differential organ preference of various tumor metastases [17].

Interestingly, this novel putative mechanism not only relates in part to a long-standing (protein deletion) theory advanced in the pre-molecular era of cancer research [22], but may also account for the increased probability of distant metastasis and extensive-stage disease correlating with poor outcome in tumor patients in which an ectopic hormone production (along with a paraneoplastic syndrome) has been ascertained [23-25].

Although this insight on a possible oncoprotein metastasis-that had been based primarily on many preceding studies on the hyperinsulinemia-cancer connection and on the presence of insulin in tumor cells-is still relatively new, there have been recent experimental reports that provide further support for this assumption. As such, the recent demonstration of the ability of the E1A viral oncoprotein to epigenetically reprogram the cells it infects [26], makes it conceivable that insulin-which shares with E1A the LXCXE RB-binding motif [27] along with the capacity to bind [28-30] and thereby inactivate RB [28]-reprograms the genes of the cells it has entered for abnormal cell proliferation. These potential insulin-induced epigenetic changes would functionally mimic both a (preceding) growth-promoting effect of an insulin-RB complex formation and a (subsequent) gene mutation pattern that may arise during the further evolution of these cells/tissues towards malignancy. In other words, the viral oncoprotein-like insulin molecule [27,31] would display two distinct properties that are functionally equivalent in terms of driving oncogenesis [18]. Moreover, the immunohistochemical identification of insulin in lung cancer tissue samples (whereby, besides the actual tumor cells, some normal pneumocytes were also revealed to be insulin-positive) in the absence of detectable insulin transcripts [32] additionally strengthens the concept of a pathological spread of (blood-borne) insulin in malignant diseases.

Beyond insulin, there are also other candidate molecules that could undergo an oncoprotein metastasis, e.g. osteopontin. Accordingly, it has been shown that osteopontin is found in premalignant and malignant cells derived from patients with tumors of the oral cavity [33] and, moreover, that osteopontin translocates to the nuclei of mitotic cells [34]. Entirely consistent with the oncoprotein metastasis concept and intriguingly, it has furthermore been shown that primary tumor-derived and blood-borne osteopontin is able to promote the microenvironmental changes necessary for distant metastatic seeds [35].

Most recently, a known amino acid labeling technique has been extended to investigate intercellular communication via both secreted and internalized proteins such as metastasis associated protein 3 and retinoblastoma binding protein 7 [36]. It will therefore be interesting to probe in future studies as to whether these proteins can add to insulin and osteopontin as mediators of the proposed oncoprotein metastasis phenomenon.

Since it thus appears that that there are various proteins that cross subcellular borders and thereby contribute to carcinogenesis, a therapeutic strategy that suggests itself in order to counteract these microbial infection-like, transcellular processes of malignancy would be to administer cell-permeable agents that directly block these mobile oncoproteins. Possible pharmacological candidates for such intervention are cell-penetrating tumor suppressor peptides, in particular those targeting the RB and nucleocrine pathways [17,18,28,30,37-40]. In this context, a parallel is noteworthy: in the same way as insulin's internalization into cells is not saturable [41] nor is that of a 16-amino acid fragment derived from the Antennapedia homeodomain and termed "Penetratin" either [42]. Hence, the use of the latter in the construction of the above-mentioned anticancer peptides [30,37,38] in order to counteract insulin-driven cell proliferation is likely to ensure (under appropriate pharmacodynamic and pharmacokinetic conditions) the achievement of a pharmacological balance which represents one of the prerequisites if oncoprotein metastasis is to be reversed.

## Implications for medicine

**Figure 2:**
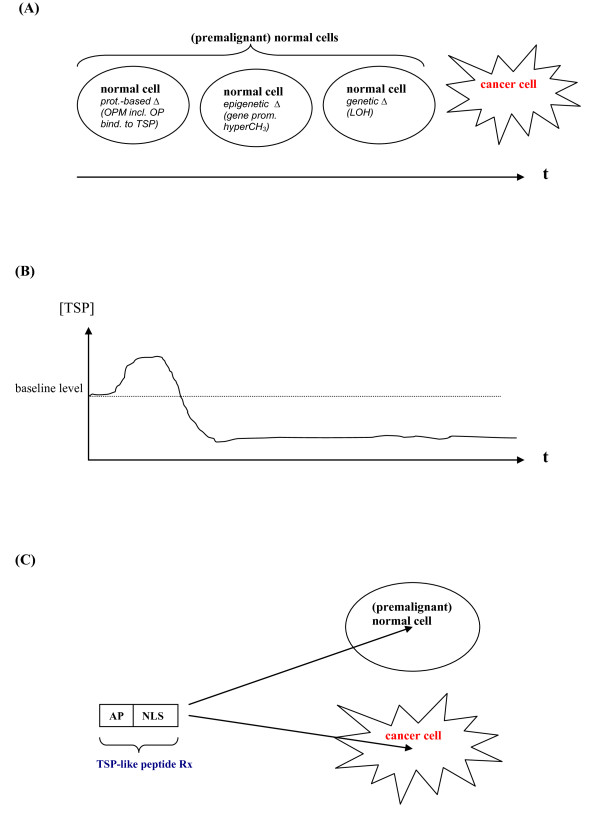
**Schematic overview of possible sequelae of oncoprotein metastasis (OPM) and a potential OPM treatment with distinct antineoplastic peptides**. a) *Morphological *sequelae of OPM and its (epi)genetic correlates ultimately making a seemingly normal cell adopt a malignant appearance ("morphological switch"). b) *Molecular *sequelae of OPM resulting in a tumor suppressor protein (TSP) loss of function (after a reactive or compensatory upsurge in response to the initial oncoprotein challenge) already at an early stage of the oncogenic process when the affected cells have still a (deceivingly) normal appearance ("functional switch"). c) *Antagonism of OPM *by treatment (Rx) with TSP-like peptides featuring a binary structure that combines an antiproliferative (AP) segment with a nuclear localization sequence (NLS) the latter of which also mediates cellular penetration/internalization and thus ensures that these antineoplastic peptides are able to enter and influence both (premalignant) normal-appearing cells and cancer cells. For a more complete picture, it should be added that non-peptide mimetics of these peptides are also conceivable (albeit, for specific reasons to be discussed elsewhere, not preferred) therapeutics. Moreover, chemopreventive (peptide and non-peptide) agents are likely to achieve their beneficial effects by a similarly global internalization into non-malignant and premalignant cells.

Taken together, I have presented additional recent evidence for the potential occurrence of oncoprotein metastasis that may be a major mechanism of premalignancy besides and/or preceding epigenetic and genetic changes in morphologically normal cells (Fig. 1b and Fig. 2a). For a complete picture it should be added that the process of oncoprotein metastasis may also occur in malignant cells and thereby contribute to their further de-differentiation.

Therefore, future studies should examine whether (morphologically) normal cells from cancer patients, in particular those adjacent to primary tumors and their metastases, i.e. pertaining to their (inflammatory) microenvironment [16], contain *oncoprotein-tumor suppressor protein heterodimers *(Fig. 1b) or, respectively, their *correlates*, e.g. posttranslational tumor suppressor protein modifications such as RB (hyper)phosphorylations [17]. For investigative purposes, this protein-based status of cancer patient-derived normal cells should be additionally compared with alike parameters of normal cells obtained from *non*-cancer patients and also from healthy individuals.

This proposed analysis, if validated, should fundamentally transform the diagnosis, prognosis and treatment of malignant disease. As previously outlined [43], such novel approach would, for instance, entail a profound revision of the surgical management of this ailment as R0 resections, i.e. tumor resections into healthy surrounding tissue, would no longer be determined by the morphology of the cells only, but also by the *subcellular *(protein-based, epigenetic and genetic) *status *of the normal-appearing cells surrounding the primary tumor and/or metastasis, respectively. The consequence therefrom would be more precise surgical resections (guided by prior subcellular analysis) which in turn should reduce the rate of local recurrence of primary tumors, e.g. of advanced stage (colo)rectal carcinomas.

Furthermore, given the loss of function of tumor suppressor proteins coinciding with an oncoprotein metastasis and its (epi)genetic correlates (Fig. 2b), drug treatment of cancer disease could equally undergo a paradigm shift through the application of cell-permeable tumor suppressor peptides that enter both morphologically normal, yet likely premalignant cells and cancer cells (Fig. 2c), as previously envisaged [17,18,39,40,44]. This potential pharmacological rationale would address not only the primary tumor, but also its distant metastases in an appropriate fashion, specifically by disrupting oncoprotein-tumor suppressor protein heterodimers and thereby reactivating tumor suppressor function in the entire organism. Hence, the survival of the cancer patient which depends primarily on the extent of successful eradication of tumor metastasis would be predictably increased.

The above-proposed therapeutic approach by means of antineoplastic, cell-permeable peptides would have bionic features as it would reflect some properties of natural molecules which combine antiproliferative properties with a propensity to shuttle in and out of cells such as interferons [39], e.g. γ-interferon [45], insulin-like growth factor binding protein (IGFBP) 3 [46,47] and the IGFBP-related HtrA1 gene product [48]. In the same way as these defensive proteins contribute to the homeostasis of cell growth, so would their artificial peptide mimetics whereby these synthetic molecules could be titrated such that the growth acceleration excess would be curtailed, yet not the entire proliferative process *per se *ablated, consistent with a previously proposed artificial induction of homeostatic defense mechanisms [49] and also a more recent view cautioning against the side effects of a complete abrogation of a given disease target [50].

## Ramifications for biophysics

**Figure 3:**
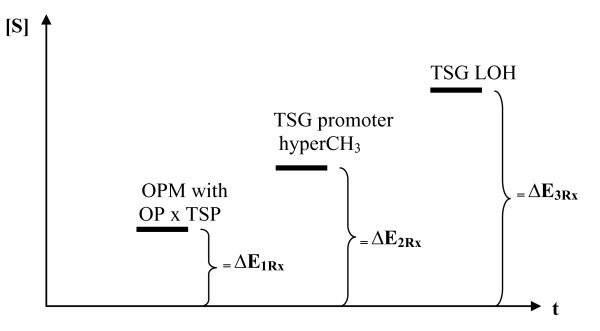
**Schematic representation of the increase in entropy (S) associated with premalignant, subcellular changes over time and its potential reversal**. More specifically, S gradually increases from the state of oncoprotein metastasis (OPM) in conjunction with oncoprotein (OP)-tumor suppressor protein (TSP) complex formations (OP × TSP) to the state of (epigenetic) tumor suppressor gene (TSG) promoter hypermethylations (hyperCH_3_) and again to the state of TSG loss of heterozygosity (LOH) defects, whereby each of their neutralization requires a corresponding amount of energy (E) or negative entropy, respectively, intrinsic to a given dose of a therapeutic compound (Rx). In this context, it should be specified that the (premalignant) stages of an OPM encompassing OP-TSP complex formations and of its epigenetic equivalent may be subject to a relatively high degree of spontaneous reversibility through natural mechanisms of cancer surveillance.

It is furthermore interesting to note that non-malignant cells in which tumor suppressor function is compromised by a) putatively oncoprotein metastasis along with oncoprotein-tumor suppressor protein complex formations, b) epigenetic silencing through hypermethylation of the promoters of tumor suppressor genes or, respectively, c) tumor suppressor gene LOH may be regarded as *(energetically) distinct quantum states of a (morphologically) normal cell *whereby an intrinsic (premalignant) evolution of this cell towards the latter state, i.e. the most entropic and thus most stable or least reversible one, could be assumed (Fig. 3). This view would thus expand on a previous biophysical concept postulating (molecular) entropy as a key driving force for carcinogenesis [51] and, moreover, be in line with observations on the (prognostically adverse) structural entropy of lung tumors [52] and the entropic accumulation of splicing defects in various carcinomas [53].

As a result, these premalignant processes might be reversed-in a dose-dependent fashion corresponding to distinct energy (or negative entropy) values (Fig. 3) - by antagonistic quantum states induced e.g. by therapeutic cell-permeable peptides in conjunction with the growth-suppressive function of endogenous proteins that these peptides may recruit through physical interactions [17,43,54]. In accordance with this view, it has been shown for a series of antineoplastic compounds including peptides that the inhibition of cell cycle progression ensuing from the disruption of protein-protein interactions requires a lower dose of the respective anticancer agent as compared to that at which (programmed) cell death (e.g. by nuclear fragmentation) occurs in cancer cells.

Moreover, the energetic or quantum states of untreated vs. treated (pre)malignant cells should be explored by physical methods, thus considerably expanding on measurements of quantum states in elements used by living systems such as shown for photosynthetic reactions [55,56]. These envisioned advances may not only be decisive for the further refinement and increased precision of diagnosis and therapy of cancer disease, e.g. by means of sequential mapping and targeting of neoplastic "fields" [5,17,51], but also further substantiate the insights of Delbrück *et al. *at the interface between biology and physics [57], ultimately making it likely that quantum biology will come of age in the foreseeable future.
